# The implementation of mindfulness-based cognitive therapy for patients with inflammatory bowel disease: a qualitative study

**DOI:** 10.1186/s40359-026-05082-4

**Published:** 2026-07-03

**Authors:** Sabien J.E. Bosman, Myrthe van der Leegte, Milou M. ter Avest, Marloes J. Huijbers, Loes H. C. Nissen, Wietske Kievit, Anne E.M. Speckens, Linda Kwakkenbos

**Affiliations:** 1 Department of Psychiatry, Expertise Centre for Mindfulness, Radboud University Medical Centre, Nijmegen, Postbus 9101, 6500 HB the Netherlands; 2 Donders Institute for Brain, Cognition, and Behaviour, Radboud University Medical Centre, Nijmegen, Netherlands; 3 Department of Gastroenterology and Hepatology, Jeroen Bosch Hospital, ‘s-Hertogenbosch, Netherlands; 4 Department of IQ Health, Radboud University Medical Centre, Nijmegen, Netherlands; 5 Department of Clinical Psychology, Behavioural Science Institute, Radboud University, Nijmegen, Netherlands

**Keywords:** mindfulness-based cognitive therapy, implementation, CFIR, barriers, facilitators, inflammatory bowel disease

## Abstract

**Background:**

Our recent trial studying mindfulness-based cognitive therapy (MBCT) for Inflammatory Bowel Disease (IBD) patients demonstrated reductions in psychological distress and improvements in well-being. Building on these findings, the present study aimed to explore barriers and facilitators for implementation and scenarios to overcome these.

**Methods:**

We conducted a sequential two-part study consisting of qualitative interviews followed by a survey. Semi-structured interviews were conducted with key stakeholders (*n* = 32) on barriers and facilitators for implementation. The Consolidated Framework for Implementation Research guided data collection and framework analysis. Based on interview outcomes, implementation scenarios were formulated and presented in a survey to stakeholders (*n* = 21) to explore their perspectives on the most suitable scenario.

**Results:**

Key barriers identified were scepticism and misconceptions about MBCT and its lack of evidence, and limited funding, time, and perceived competency to discuss mental health in medical consultations. Facilitators included the presence of a local champion, growing attention to lifestyle factors in society and healthcare, and the group-based design of MBCT providing peer support. Stakeholders preferred integrating MBCT within a regional network, but opinions differed. Irrespective of the preferred scenario, stakeholders emphasized the need for a strong business case, including evidence on long-term (cost-)effectiveness, to secure broad support and insurance coverage.

**Conclusions:**

Although implementing MBCT for IBD patients is worthwhile, several prominent barriers currently hinder this process. A strong business case is needed to gain broad support and insurance coverage. Further research is required to determine the most suitable and feasible implementation scenario and associated strategies.

**Clinical trial number:**

not applicable.

**Supplementary Information:**

The online version contains supplementary material available at https://doi.org/10.1186/s40359-026-05082-4.

## Background

Patients with Inflammatory Bowel Disease (IBD), including mainly Crohn’s disease and ulcerative colitis, suffer from poor physical functioning, fatigue, and sleep disturbances even when the IBD is in remission [1]. In addition, many of them experience psychological distress, including symptoms of depression (25.2%) and anxiety (32.1%) [2], resulting in a lower quality of life, poorer self-care, and more healthcare use [3–5]. Therapeutic strategies primarily involve medication use to control physical symptoms [6]. However, mental health interventions are needed to improve quality of life and reduce psychological distress in IBD patients.

A promising psychological intervention for IBD patients is Mindfulness-Based Cognitive Therapy (MBCT), an eight-week group-based intervention, consisting of mindfulness-based meditative practices and cognitive therapy [7]. It teaches patients to be aware of thoughts, emotions, and bodily sensations in the present moment in a kind, investigative and non-judgemental way. A large umbrella meta-analysis of 44 meta-analyses has shown that MBCT is effective in reducing psychological distress and improving quality of life in patients with various psychiatric and somatic diseases [8].

The evidence of MBCT for IBD patients specifically is growing. A meta-analysis of eight randomised controlled trials showed positive results of mindfulness-based interventions on stress, depression, and quality of life [9]. Our recent MindIBD trial evaluating the effectiveness of MBCT for quiescent IBD patients showed that participants in the MBCT + treatment as usual (TAU) group reported less psychological distress (*d* = 0.61) and improved well-being (*d* = 0.41) post-intervention compared to those receiving TAU [10]. Moreover, MBCT had a probability of 59.8% and 72.5% to be cost-effective from a societal and healthcare perspective, respectively [11]. Participants were largely positive about MBCT, which was considered both feasible and acceptable [12]. Given these positive findings on (cost-)effectiveness and feasibility, implementing MBCT into clinical practice seems justified. However, implementation of health interventions in care settings is often difficult. Even when abundant evidence exists, it takes on average 17 years for 14% of original research findings to get translated to patient care [13]. An important first step in implementation science is the identification of barriers and facilitators to implementation [14].

No studies have examined the barriers and facilitators for the implementation of MBCT for IBD patients yet. The few studies that have been done on the implementation of MBCT have shown that implementation is difficult due to structural, cultural, and resource-related barriers, such as budgetary constraints, a low priority for MBCT by healthcare professionals, and dependence on enthusiastic and committed individuals in combination with lack of managerial support [15–18]. This study aimed to examine barriers and facilitators of implementation of MBCT for IBD patients in particular and explored stakeholders’ preferred scenarios for integration of MBCT into clinical care. This study focuses on IBD care as a specific example of a chronic somatic condition in which providing psychological support in addition to medical care is increasingly recognized as important, yet not routinely integrated into standard care.

## Methods

The MindIBD study was approved by the ethical review board CMO Arnhem-Nijmegen (#2021–7319; NL75762.091.20). Participants provided written informed consent. The Standards for Reporting Qualitative Research were used to structure and report this study [19].

### Part 1: in-depth qualitative interviews

Between July 2023 and March 2024, individual, semi-structured interviews were conducted with key stakeholders, including healthcare insurers, representatives of professional associations, representatives of the patient association, hospital healthcare procurement, managers, healthcare professionals, and mindfulness teachers to identify facilitators and barriers for the implementation of MBCT for IBD patients in clinical practice.

A purposive sampling strategy was used to recruit stakeholders with relevant expertise in IBD care and/or MBCT implementation. The research team’s professional network was used as a starting point to identify individuals with appropriate experience and roles in clinical practice and implementation. Stakeholders were invited via email to participate in an in-depth interview using a semi-structured topic guide (Additional file 1) based on the Consolidated Framework for Implementation Research (CFIR) [20]. CFIR is a practical framework designed to help guide systematic assessment of facilitators and barriers influencing the implementation of an intervention consisting of five main domains: intervention characteristics, outer domain, inner domain, individual domain, and implementation process domain. Innovation characteristics focused on the intervention itself, i.e., MBCT. The outer setting domain included external factors such as societal influences, Dutch governmental laws and policies, and financing by insurance companies. The inner setting domain concerned the direct healthcare delivery setting, such as hospitals and general practices. The individual characteristics captured healthcare professionals’ and patients’ beliefs and perspectives that influence the implementation process. Since this study was performed pre-implementation, the implementation process domain focused on the perceived needs and preconditions for implementation.

All interviews were conducted by two interviewers, a PhD-student (SB) joined by one of six other researchers, another PhD student (MtA) and five senior researchers (WK, LN, MH, EB, AS). An intern (MvdL) observed seven interviews. All interviewers were female, except one. Most interviews were conducted with one stakeholder, but four interviews were conducted with two. Interviews were conducted in Dutch, performed via videoconferencing, audio-recorded, and transcribed verbatim. Summaries of the interviews were shared with stakeholders via email to verify interpretation of the findings [21]. In case of misinterpretation, this was added to the transcripts to ensure correct interpretation.

A deductive framework analysis approach was used to map barriers and facilitators to the CFIR [22]. The CFIR constructs and definitions as described by the CFIR Leadership Team were adopted to guide analysis [23]. Coding was performed using Atlas.ti (version 24.0.0.29576). If data did not align with the framework, new codes were introduced. To enhance reliability, two coders (SB, MvdL) independently coded the first five transcripts and compared and discussed codes until consensus was reached. The remaining transcripts were coded by one researcher (MvdL), with another reviewing the codes (SB). Lastly, the research team discussed and refined the mapping of the codes onto the CFIR framework.

### Part 2: scenario survey

The second part of this study involved a survey designed to explore the most suitable setting to integrate MBCT into clinical care for IBD patients (Additional file 2). Interviews revealed that there was no consensus on the most suitable place for MBCT in the IBD care pathway. Based on these insights, we developed four potential implementation scenarios that may tackle the different barriers identified: MBCT being offered in primary care, in secondary care, within a regional network, or in the social domain. These four scenarios were explained in detail in the survey (Additional file 2) and are briefly explained below.

- When MBCT is provided in primary care, the general practice-mental health professional provides it. MBCT is available to all patients with chronic somatic diseases, such as IBD, Chronic Obstructive Pulmonary Disease, rheumatic diseases, Multiple Sclerosis, chronic pain, etc.
- When MBCT is provided in secondary care, it is organised and delivered within the hospital. Two possibilities arose: (1) providing MBCT training by a qualified mindfulness teacher employed by the hospital or (2) providing MBCT training by a member of the department of medical psychology with gastroenterologists and/or IBD nurses referring patients. It is possible to provide MBCT to a homogeneous group of IBD patients or to a broader patient population.
- When MBCT is provided in a regional network, gastroenterologists, IBD nurses, and general practitioners can refer IBD patients to qualified mindfulness teachers in the region. Patients can also choose to participate in an MBCT training without referral from their healthcare professional. In this scenario, the role of the mindfulness teacher is comparable to that of a dietician or physiotherapist [24]. In this scenario, it is possible to either provide MBCT to a homogeneous group of IBD patients or to a broader (patient) population.
- When MBCT is provided in the social domain, it is offered outside of the healthcare setting. It can be offered by employers, educational institutes, or municipalities, for example. In this scenario, MBCT is available to a wider population.

Scenarios were presented to all interviewed stakeholders via a survey that was distributed in May 2024 via the online platform CastorEDC [25]. Stakeholders were asked to assess the implementation scenarios on suitability via a five-point Likert-scale (1 = highly unsuitable, 2 = fairly unsuitable, 3 = neutral, 4 = fairly suitable, 5 = highly suitable) and to rank the scenarios according to their preference. An optional open text field was provided for each scenario to share perspectives. Scenarios were presented in a random order to minimise the potential priming effect of sequence bias [26]. Respondents received a reminder email in case of no response.

Descriptive statistics were used to present survey results, including means and frequency distributions to provide insight into the preferences on the implementation scenarios. Answers to the open-field questions were reviewed and summarised, highlighting common ideas and patterns across answers by two independent researchers.

## Results

### In-depth qualitative interviews

A total of 32 stakeholders participated in 28 interviews (Table 1). All codes with an example quotation mapped onto the subdomains of the CFIR are presented in Additional file 3. All the stakeholders agreed with the interpretations in the member check. Data saturation was achieved.

**Table 1:** Number of stakeholders per stakeholder group that participated in interviews and survey

Stakeholder group	Interviews (*n*)	Survey (*n*)
Gastroenterologists	5	3
IBD nurses	2	2
Mindfulness teachers	2	2
Medical psychologists	3	2
General practitioners	3	3
Hospital healthcare procurement	2	1
Patient representative	2	1
Healthcare insurer	3	1
Representative of professional associations in field of IBD or mindfulness	8	6
Department manager	1	0
Healthcare innovator	1	0

#### Innovation characteristics

Scientific evidence on the effectiveness of MBCT for IBD patients was considered a key factor in its implementation. Some representatives from professional associations viewed the existing evidence for MBCT in somatic diseases, including IBD, as sufficient and mentioned it as a facilitator. Most interviewees, however, expressed concern about current evidence on the long-term (cost-)effectiveness of MBCT specifically for IBD patients, representing a barrier. Another barrier mentioned by medical psychologists was the abundance of treatment options, making it unclear which treatment is most suitable. Additionally, the extensive and expensive requirements to become a certified mindfulness teacher were considered a barrier.

*“[*Interviewer: You mentioned that MBCT is currently not part of the clinical care pathway. Do you have any idea why that is?*] Yes*,* I think because the ‘evidence’ has not been sufficiently proven yet*,* it seems to me.” – Gastroenterologist*

Most stakeholders identified the group-based format as facilitating, primarily because it fosters peer support. Moreover, having a teacher with a background in (mental) healthcare was seen as helpful, as such teachers were perceived to be more competent and trustworthy.

#### Outer setting

Barriers within the outer setting domain included scepticism, misconceptions, and negative perceptions of mindfulness. Stakeholders stated that some (healthcare) professionals and policy makers do not regard mindfulness as evidence-based treatment. A few questioned whether MBCT should be considered part of healthcare at all.

*“[*Respondent explains perceived importance of psychosocial interventions, including MBCT*] Yes*,* and then some gastroenterologists simply say yes*,* big nonsense” – IBD nurse*

In addition, stakeholders noted that MBCT is not routinely reimbursed by healthcare insurance, and therefore healthcare professionals are reluctant to offer it. Several stakeholders, particularly those with an economic background, warned that there is little to no financial capacity to fund additional interventions, because healthcare budgets are fixed and limited. Consequently, they questioned what existing care would need to be discontinued to make room for MBCT. Lastly, the organisation, funding, and regulatory structure of the Dutch healthcare system was mentioned as a barrier to innovation in general. One healthcare insurer noted that this structure does not align with our innovative capacity and pace, as the National Health Care Institute can assess only one or two innovations per year for insurance coverage, while many more innovations are being developed and are seeking reimbursement.

*“Because if we pay for this mindfulness training with healthcare funds*,* what [*current care*] are we going to stop? It’s not that the budget increases.” – Healthcare insurer*

Facilitators were also mentioned in the outer setting, including the general positive perception and popularity of mindfulness in society, reinforced by increased attention through social media. Stakeholders further noted a broader societal trend toward lifestyle-oriented approaches, which aligns well with the core principles of mindfulness. This trend is also evident in healthcare, where lifestyle factors and non-pharmacological treatment options are increasingly addressed during care consultations within hospital settings, including in IBD care. Finally, representatives from a professional association of mindfulness teachers indicated that enough qualified mindfulness teachers are available nationwide to deliver MBCT.

*“And there is currently much more attention to quality of life within IBD care. […] So*,* it wouldn’t surprise me at all that this* [MBCT] *will also be included in this [*focus on quality of life and lifestyle*]” – Gastroenterologist*

#### Inner setting

Within the inner setting domain, the main barrier identified was the high workload and limited time of healthcare professionals to address the mental health needs of patients. Moreover, healthcare professionals were perceived to prioritize physical health over mental health during care consultations. The same was seen in policy makers, illustrated by the example of phasing out of medical psychology services in their hospital. In addition, healthcare professionals often lacked knowledge about referral options for MBCT and did not have access to clear information on available services. Finally, practical barriers included a lack of a suitable space, and a shortage of healthcare professionals qualified to teach MBCT within clinical settings.

*“… there are also a lot of processing problems and existential problems*,* life stage problems that can occur with any chronic condition […] These are the areas we often neglect as practitioners.” – Professional associations*

The main facilitator mentioned during the interviews was the presence of an enthusiastic and driven local champion, who took the lead in providing and implementing MBCT. Another facilitator was that healthcare professionals, particularly IBD nurses, viewed addressing patients’ mental health as an integral part of their role. Moreover, the digitalization of pre-consultation questionnaires was seen as a facilitator, making consultations more efficient, thereby creating more time to discuss topics such as mindfulness. Finally, implementation was further supported when the vision of care settings (i.e., the organizational approach to care) emphasized enhancing overall well-being rather than treating disease alone.

*“So that’s the advantage*,* that it’s a nursing theme. And that care belongs with them.” – IBD-nurse*

#### Individual domain

##### Healthcare professionals

Stakeholders believed that healthcare professionals, especially gastroenterologists, were not fully convinced of the need of an intervention such as MBCT within care settings. Moreover, most healthcare professionals did not feel sufficiently competent or able to discuss mental health issues, given the limited time available during consultations. General practitioners noted that they had little opportunity to address these issues, because IBD is mainly organized in secondary care.

“*Just like IBD patient groups who experience substantial psychological burden from their illness*,* most find it very nice and useful if attention is paid to this. We just don’t like doing it [*discussing mental health*] ourselves.” – Professional association*

Although most stakeholders acknowledged the aforementioned scepticism and misconceptions within society and among their colleagues, they themselves were generally positive and believed MBCT could address an important unmet need. Some also mentioned that an increasing number of their colleagues shared this view or were beginning to do so. Moreover, IBD nurses and general practitioners felt sufficiently competent to discuss mental health with patients.

*“I think that we all have increasing attention for the impact psyche can have on somatic complaints. So*,* in that way I definitely feel competent to start that conversation” – General practitioner*

##### Patients

Stakeholders mentioned that not all patients are receptive to mindfulness; some may be sceptical, and others may perceive psychological treatment as inappropriate for a somatic disease. Some stakeholders suggested that a strong therapeutic relationship between the healthcare professional and patient could help increase the acceptability of MBCT for patients.

At the same time, some stakeholders reported positive attitudes of patients toward psychological interventions, including mindfulness. Several noted that an increasing number of patients are actively inquiring about non-pharmacological treatment options and are open to mindfulness-based approaches. One patient representative stated that some patients would be willing to pay for MBCT themselves. Lastly, the specific adaptations to IBD patients made in the MindIBD trial were considered facilitating.

“*Yes, that *[enthusiasm for MBCT] *will be very different. If you have the down-to-earth guy, he might think: what are you talking about?” – IBD nurse*

#### Implementation process

Several perceived needs and preconditions for implementation emerged during the interviews. Stakeholders agreed that broad support for MBCT is essential, including support from patients, healthcare professionals, professional associations, and policy makers. Moreover, most stakeholders emphasized that MBCT should be covered by healthcare insurance. To achieve both broad support and insurance coverage, stakeholders highlighted the importance of a strong business case. Such a business case should include robust scientific evidence demonstrating (long-term) (cost-)-effectiveness, societal impact, improvements in quality of life, and feasibility.

*“That still requires evidence*,* and I think it requires stories*,* especially from patients who participated and talk about what it has done for them. […] Yes*,* that would be fantastic*,* of course. Yes*,* if you can demonstrate a business case” – Mindfulness teacher*

Although stakeholders expressed positive attitudes toward MBCT for IBD patients, it became clear from the interviews that no single stakeholder group felt they were in the lead for its implementation, and there was no consensus on where MBCT should be positioned in IBD care. Some stakeholders favoured implementation within secondary care, others within primary care or the social domain, and others preferred a regional network approach.

### Part 2: scenario survey

Of the 32 interviewed stakeholders who were invited to rate the implementation scenarios, 21 (65.6%) responded (Table 1). In addition to providing scores (Table 2), approximately half of the stakeholders also left brief notes on the scenarios, depending on the scenario (range *n* = 8 to *n* = 14).

**Table 2:** Suitability scores for the implementation scenarios

Implementation scenario	Suitability mean (range)	Preference *n* (%)*
MBCT offered in a regional network	4.48 (3–5)	9 (45%)
MBCT offered in primary care	3.74 (1–5)	5 (25%)
MBCT offered in secondary care	3.90 (2–5)	4 (20%)
MBCT offered in the social domain	3.11 (2–5)	1 (5%)

*One of the respondents did not rate MBCT in primary care and in secondary care and did not rank the scenarios

Respondents preferred MBCT being offered in a regional network (*n* = 9; 45%) (Table 2). They liked the accessibility for patients, because of limited travel time and availability of MBCT for multiple chronic diseases, while a regional network can ensure quality. It was also mentioned that this would fit into the national trend towards care networks. A counterargument raised was that you might lose the connection to IBD.

Five stakeholders opted for MBCT being offered in primary care (25%), as they felt it was the most accessible option and could reach a broader patient population. However, concerns were also raised: general practices and their mental health professionals are already experiencing a (too) high workload. One of the respondents also mentioned they did not believe general practitioners are responsible for this type of care.

Four stakeholders (20%) preferred MBCT being offered in secondary care arguing that referral to MBCT within the hospital is easy, and this scenario also makes it possible to provide MBCT for other patient groups. In addition, peer support can be arranged, and it is a familiar environment for patients. However, some of the respondents were against this scenario since it would increase the already high burden on hospitals. They also mentioned the lack of capacity and the greater travel distance for patients. Some respondents expressed the view that hospitals should not be responsible for delivering this type of care.

One participant, a healthcare procurer, favoured MBCT being offered in the social domain (5%), as they were not convinced MBCT should be medicalised given the constraints of limited personnel and budget. Main arguments against MBCT in the social domain were the potential loss of disease-specific adaptations, and the patients’ dependence on regional and municipal resources, which may result in inequalities in access.

## Discussion

This study identified important barriers and facilitators for the implementation of MBCT for IBD patients using the CFIR framework and explored preferred implementation scenarios. Main barriers were scepticism and misconceptions about MBCT for IBD and its lack of evidence, limited funding, time, and competencies to discuss mental health in care consultations, and lack of a responsible party to drive implementation. Main facilitators were the presence of a champion, growing attention to lifestyle within society and healthcare, and the group-based design that can facilitate peer support.

The survey provided insight into how these barriers and facilitators can be translated to daily clinical practice and gave direction to the next possible steps in implementation. There was no consensus among stakeholders on the best scenario for integrating MBCT in healthcare, but survey results indicated a preference for offering MBCT in a regional network. A regional network is thought to increase availability, while ensuring quality, capacity, and sustainability, and thereby be a solution to several barriers found in the interviews, particularly in the inner setting CFIR domain, such as limited time and competencies. However, there were substantial differences between respondents with some of them preferring to offer MBCT within healthcare, while others were questioning this. Regardless of the delivery scenario, stakeholders emphasized the need to a strong business case. The business case should describe the specific funding challenges associated with the chosen scenario. In case of a regional network, it is important to note that financing MBCT does not fit in the current reimbursement system for IBD care. Therefore, the business case should provide guidance on alternative ways to cover costs, such as patients paying for (some) costs themselves, or integrating the cost of MBCT into medical care covered by healthcare insurers.

Our study showed that stakeholders were generally positive and supportive of the implementation of MBCT for IBD patients. However, key barriers need to be addressed first. These barriers are widely recognised in other literature. Our study found a large discrepancy between the existing evidence for MBCT for IBD patients and the perception of healthcare professionals of this evidence, indicating a gap in knowledge translation. Limited awareness among healthcare professionals of the existing evidence is a recognised challenge in the implementation of integrative medicine, such as MBCT [27–31], which is an important prerequisite in implementation science [14]. Other studies have also found mixed results regarding healthcare professionals’ perspectives on the usefulness of integrative medicine, such as MBCT [27, 28]. Moreover, they found that many healthcare professionals feel insufficiently equipped to discuss integrative medicine because of limited knowledge and training. Additionally, the lack of stable funding we identified reflects a more widely recognised challenge in the implementation of similar interventions [27, 32]. Lastly, the absence of champions is often described in literature as an important barrier in implementation efforts [17, 32, 33]. These barriers are starting points for the development of implementation strategies, which is the next step according to implementation research [14].

To implement MBCT for IBD patients, several evidence-informed implementation strategies can be proposed for the beforementioned barriers based on the CFIR-ERIC taxonomy [34]. This includes, among other strategies, the development of a business case, which is a document that substantiates the value of implementation by weighing costs, benefits, and risks with input from IBD-specific (cost-)effectiveness research. It can positively influence implementation and gain stable funding [27, 34, 35]. Knowledge, support, and attitudes towards MBCT should be improved by increasing knowledge through training and educational outreach visits [34, 36]. To support such efforts, coordinated action and long-term stakeholder commitment are required, which can be supported by a coalition of interprofessional stakeholders and local champions [32, 34]. Since none of the stakeholders was eager to take sole responsibility for implementation efforts and no consensus existed on the most suitable place for MBCT in the IBD care pathway, more research is necessary on the most suitable and feasible implementation scenario and the associated implementation strategies. Possible responsible parties could include the professional associations of either mindfulness trainers or IBD-related healthcare professionals, including gastroenterologists, IBD-nurses, or medical psychologists.

### Strengths and limitations

To our knowledge, this is the first study that explored the perspectives of stakeholders on the barriers and facilitators of the implementation of MBCT for IBD patients. It can be seen as a specific example of implementing MBCT within somatic care. Further research, also in other somatic diseases, can build on our results. A notable strength is that it included stakeholders from several relevant stakeholder groups. However, the recruitment strategy may have resulted in an overrepresentation of stakeholders who are familiar with or positively disposed toward MBCT, potentially contributing to the high levels of perceived acceptability reported in this study. As a result, critical or less favourable perspectives on MBCT implementation may be underrepresented. We have tried to mitigate this by explicitly stating in the invitation and in the interviews that we were in interested in all possible perspectives, also negative ones. Moreover, for some stakeholder groups, including IBD nurses, patients, and insurers, only a few participants were included in the interview and survey samples, which may have resulted in an incomplete understanding of their perspectives. Another limitation is the limited number of stakeholders who responded to the survey, which might have compromised the generalisability of the results. Therefore, this should be interpreted as a first exploration of the perspectives on the best implementation scenario and more research is needed to provide a more solid understanding of the scenario that would be most widely accepted and supported. Lastly, our results might be less generalisable to other countries with different reimbursement structures, workforce capacities, or levels of integration of MBCT into care settings. While some identified barriers and facilitators, such as misconceptions, limited time, and limited competencies are likely to be relevant across healthcare systems, factors related to financing and organizational structure may limit direct transferability to fee-for-service systems (like the US) or fully socialized systems (like the UK NHS). Additional exploration of barriers and facilitating in the CFIR outer setting domain may be warranted in these contexts.

## Supplementary Information

Supplementary Material 1. Additional file 1.docx → Interview topic guide.

Supplementary Material 2. Additional file 2.docx → Survey on implementation scenarios.

Supplementary Material 3. Additional file 3.docx → Codes with an example quotation mapped onto the subdomains of the CFIR framework.

## Data Availability

The data used and/or analysed during the current study are available from the corresponding author on reasonable request.

## Abbreviations

CFIR: Consolidated Framework for Implementation Research
CFIR-ERIC: Consolidated Framework for Implementation Research – Expert Recommendations for Implementation Change
IBD: Inflammatory Bowel Disease
MBCT: Mindfulness-based cognitive therapy
PhD: Doctor of Philosophy
TAU: Treatment as Usual
