# Supplementary material for: The implementation of mindfulness-based cognitive therapy for patients with inflammatory bowel disease: a qualitative study
Source: BMC Psychol. 2026 Jul 3;14:1377. doi: 10.1186/s40359-026-05082-4 (PMC13599151; doi:10.1186/s40359-026-05082-4)
Supplement: Supplementary file 1 [file 40359_2026_5082_MOESM1_ESM.docx]

# Additional file 1: Interview topic guide

Welcome and Introduction

Thank you very much for participating in this interview.

We are currently working on the MindIBD project: a study into the effects of mindfulness in patients with Crohn's disease or Ulcerative Colitis. In this project, we aim to improve coping strategies, which may benefit people over many years of treatment. Our ultimate goal is to implement mindfulness into the care of patients with IBD. To achieve this, we are conducting a process evaluation, in which we are engaging with all key stakeholders to discuss both the barriers and facilitators to implementing mindfulness in IBD care.

All data collected during this interview will be processed anonymously and will only be used for data analysis related to this research. For this purpose, it is important that the interview is recorded, so we can transcribe and analyse it. Do you consent to this?

[Starting the recording (and mention the date)]

Do you have any questions before we begin?

*Introducing the interviewers*

Introduction

1. Could you please introduce yourself?

Part 1: Personal Opinion and Experiences with Mindfulness

2. To what extent are you familiar with mindfulness?
3. What are your thoughts on the idea of offering mindfulness to patients with IBD?
   a. Why is that?

Part 2: Implementation

1. What factors could contribute to the implementation of mindfulness in IBD care?
2. What barriers do you foresee in the implementation of mindfulness for IBD patients?
3. How would you implement mindfulness for IBD patients in practice?

Topics – including probes – based on the CFIR model:

**I. Intervention Characteristics**

1. **Innovation evidence-base** – How do you view the current evidence for mindfulness in IBD?
2. **Innovation relative advantage** – How do you view mindfulness in comparison to other existing interventions? What are the advantages and disadvantages?
3. **Innovation trialability** – How do you see the adaptability of mindfulness training? How important is this for IBD patients?
4. **Innovation costs** – How do you view the costs of mindfulness for IBD patients?
5. Innovation source
6. Innovation adaptability
7. Innovation complexity
8. Innovation design

**II. External Environment**

1. **Local attitudes** – How do you think mindfulness for IBD will be viewed by the environment, such as patients or other healthcare providers?
2. **Partnerships & connections** – How do you view collaborations between mindfulness trainers and IBD healthcare providers? How are partnerships formed with other parties?
3. **Policies & laws** – How do you view the qualifications required for mindfulness trainers to work with groups of IBD patients?
4. **External pressure** – To what extent do you feel pressure to implement mindfulness for IBD patients, and where does that pressure come from?
5. **Financing** – What financial or other incentives currently exist to either implement or not implement mindfulness? What financial or other incentives could contribute to the implementation of mindfulness in IBD care?
6. Critical incidents
7. Local conditions
8. External pressure
   a. Societal pressure
   b. Market pressure
   c. Performance measurement pressure

**III. Internal Environment** – How can your organisation contribute to the implementation of mindfulness for IBD?

1. **Structural characteristics** – How can the infrastructure of your organisation influence the implementation of mindfulness for IBD patients? (Here, examples can be mentioned, such as referral processes, workflow, information dissemination points, staff roles, etc.)
2. **Relational connections** – How is collaboration with other departments within your organisation?
3. **Communications** – How is information shared and how does communication flow within your organisation?
4. **Culture** – How do you think the culture within your organisation will impact the implementation of mindfulness for IBD patients?
5. **Relative priority** – How do you view the priority of implementing mindfulness for IBD compared to other innovations?
6. **Mission alignment** – How does the implementation of mindfulness align with the vision of your organisation?
7. **Available resources** – What is (or isn’t) available for the implementation of mindfulness for IBD? Consider resources such as space, informational materials, etc.
8. **Access to knowledge & information** – To what extent are you supported in implementing mindfulness? How would you like to be supported?
9. Tension for change
10. Incentive systems

**IV. Individual Characteristics** – How do you view the implementation of mindfulness for IBD?

1. **Opinion leaders** – How do individual people influence the implementation of mindfulness for IBD? What type of individuals are these?
2. **Need** – How do you view the need for mindfulness in IBD? Think about the needs of patients or healthcare providers.
3. **Capability** – How do you view your own skills and knowledge regarding mindfulness in the context of its implementation?
4. **Opportunity** – How feasible is it for you to play a role in the implementation of mindfulness for IBD?
5. **Motivation** – How motivated are you to play a role in the implementation of mindfulness for IBD?

**V. Process – How would you implement mindfulness for IBD patients in practice? (Responsibility, which organisations are involved)**

1. Teaming
2. Assessing needs
   1. Innovation deliverers
   2. Innovation reciepients
3. Assessing context
4. Planning
5. Tailoring strategies
6. Engaging
   1. Innovation deliverers
   2. Innovation recipients
7. Doing
8. Reflecting & evaluating
   1. Implemention
   2. Innovation
9. Adapting

Acknowledgements and closing

- Summary of the points discussed
- Is there anything else you would like to add to this interview?
- Thank you for your participation
- A summary will be sent to you for review
