# Supplementary material for: The implementation of mindfulness-based cognitive therapy for patients with inflammatory bowel disease: a qualitative study
Source: BMC Psychol. 2026 Jul 3;14:1377. doi: 10.1186/s40359-026-05082-4 (PMC13599151; doi:10.1186/s40359-026-05082-4)
Supplement: Supplementary file 2 [file 40359_2026_5082_MOESM2_ESM.docx]

# Additional file 2: Survey on implementation scenarios

Thank you very much for taking the time to complete this survey. Your involvement in this research has already been very valuable so far.

We are investigating whether mindfulness can help patients with Crohn’s disease or ulcerative colitis (collectively known as IBD) in improving mental health issues, fatigue, and sleep. In this final part of the project, we are looking for the best way(s) to implement mindfulness into the care for IBD patients. For this, we have conducted 28 interviews with various experts, including gastroenterologists, IBD nurses, general practitioners, medical psychologists, mindfulness trainers, representatives from the patient federation and professional associations, policymakers, managers, and health insurers. We now have a clearer understanding of the factors that hinder or facilitate implementation.

Currently, mindfulness is not reimbursed for people with physical conditions within the healthcare system. Mindfulness is only covered under the basic insurance package for patients who meet the criteria for a psychiatric disorder. Some health insurers include it in supplementary insurance for burnout symptoms. Based on the interviews, we have developed four possible implementation scenarios along with appropriate financing options for people with IBD or other chronic physical conditions.

We would like to ask you, as an expert, to reflect on these scenarios from your perspective with a few questions. This will take approximately 15 minutes of your time.

The survey is an important part of our research on the implementation of mindfulness for people with chronic diseases, and your input is of great importance. All data collected through this survey will only be used for this research.

**Scenario 1: Regional Network**

In a regional network, mindfulness would be offered in a similar way to physiotherapy and dietetics within the current healthcare system. This means patients could be referred to a mindfulness practice or trainer in their own region by their healthcare provider (such as gastroenterologists, IBD nurses, and GPs). Additionally, it might be possible for patients to independently choose mindfulness training, potentially after a conversation with their healthcare provider. A referral would not be required for patients.

For this scenario, it is essential that there are a local network and coordination between mindfulness practices/trainers and healthcare providers. In this scenario, mindfulness could also be relatively easily offered to patients beyond just those with IBD, such as patients with other chronic diseases (e.g., COPD, rheumatic diseases, MS, irritable bowel syndrome, etc.)

*How suitable do you think this scenario is for offering mindfulness to IBD patients?*

1 – Not suitable; 2 – Quite unsuitable; 3 – Neutral; 4 – Quite suitable; 5 – Very suitable

*Could you provide an explanation? (optional)*

**Scenario 2: Hospital care**

In this scenario, mindfulness is organised within the hospital setting. The training would take place in the hospital. For this, the hospital would need to hire a mindfulness trainer if one is not already present, or staff already working within the hospital could be trained (such as IBD nurses with an interest in mindfulness). Mindfulness training could also be provided by another department, such as medical psychology. In this case, the gastroenterologist or IBD nurse would refer the patient to the medical psychology department.

For this scenario, it is essential that the hospital, in addition to having qualified mindfulness trainers, also has a suitable space for the training. Close collaboration between the gastroenterology department and the medical psychology department is important. This scenario offers the opportunity to form specific groups of IBD patients, which could provide valuable peer support.

*How suitable do you think this scenario is for offering mindfulness to IBD patients?*

1 – Not suitable; 2 – Quite unsuitable; 3 – Neutral; 4 – Quite suitable; 5 – Very suitable

*Could you provide an explanation? (optional)*

**Scenario 3: General Practitioner / General practice-mental health professional**

In this scenario, mindfulness training is offered by general practice surgeries. General practice-mental health professional would provide these training sessions for patients in their practice. GPs could offer this to IBD patients. Gastroenterologists and IBD nurses could also bring up mindfulness with IBD patients and refer them to the GP for specific training.

For this scenario, it is possible to offer training not only to IBD patients but also to patients with other chronic conditions, such as COPD, rheumatoid arthritis, MS, chronic pain, etc.

*How suitable do you think this scenario is for offering mindfulness to IBD patients?*

1 – Not suitable; 2 – Quite unsuitable; 3 – Neutral; 4 – Quite suitable; 5 – Very suitable

*Could you provide an explanation? (optional)*

**Scenario 4: Social domain**

In this scenario, mindfulness is offered outside the healthcare setting. It could be provided by, for example, the employer, an educational institution, or the local municipality. In this scenario, the healthcare provider could have a role in making mindfulness a topic of discussion for IBD patients and informing them about qualified trainers in the region. Mindfulness would not specifically be offered to IBD patients, but to anyone who might benefit from it.

*How suitable do you think this scenario is for offering mindfulness to IBD patients?*

1 – Not suitable; 2 – Quite unsuitable; 3 – Neutral; 4 – Quite suitable; 5 – Very suitable

*Could you provide an explanation? (optional)*

**Conclusion: These questions concern all scenarios**

*If you had to rank the scenarios in order of preference, with the first being your most preferred and the last being your least preferred, what would that order be? Choose from: General Practitioner / POH-GGZ, Regional Network, Social Domain, Secondary Care*

*Could you provide an explanation?*

**Thank you very much for your time and effort in completing this survey.**
