# Supplementary material for: The implementation of mindfulness-based cognitive therapy for patients with inflammatory bowel disease: a qualitative study
Source: BMC Psychol. 2026 Jul 3;14:1377. doi: 10.1186/s40359-026-05082-4 (PMC13599151; doi:10.1186/s40359-026-05082-4)
Supplement: Supplementary file 3 [file 40359_2026_5082_MOESM3_ESM.docx]

# Additional file 3: Codes with an example quotation mapped onto the subdomains of the CFIR framework

| **Domain** | **Barriers** | **Facilitators** |
| --- | --- | --- |
| **I. Innovation domain** |  |  |
| Innovation source | **Professional background of teacher not in (mental) healthcare hampers connections with healthcare**  *“But that's also kind of what your background is. Look, of course I have—I don't have a mental health background or not—And what I really—I once called a GP and he said yes, but are you a psychologist? And then I said no. Well, too bad you know. And then you're right—Hey, because when I say I have 20—They look at my LinkedIn, yes, 20 years of accountancy, yes, you know, bye.” - Professional association* | **Professional background of teacher inside (mental) healthcare important for connections with healthcare**  *“Yes, because I notice that people who are already in mental health care, for example, flow back and forth in that world much more easily because they already know people.” - Professional association* |
| Innovation evidence-base | **Insufficient scientific evidence (for IBD)**  *“Yes, I think because the 'evidence' has not yet been sufficiently proven, it seems to me.” - Gastroenterologist* | **Scientific evidence of mindfulness for somatic symptoms**  *“There are of course studies into somatic symptoms. And it turns out that mindfulness simply helps.” - Professional association* |
| Innovation design |  | **Group-based design (with only IBD / peer support)**  *“But the participants, when we talk about patients, find it very valuable that they are in a homogeneous group” - Mindfulness teacher* |
| Innovation relative advantage | **Number of treatments offered for mental health**  *“There is so much that you sometimes don't know, what should I offer someone?” - Medical psychologist* |  |
| Innovation complexity | **Requirements for a qualified mindfulness trainer.**  *“And what I understood was that you also have to be a registered mindfulness trainer to be able to offer that mindfulness course and we actually had no colleagues within our department at that time who had the affinity to do the entire training with all the re-registration requirements that come with it, because they are really quite hefty. So it is difficult to offer that from medical psychology, because then you do not have that registration.” - Medical psychologist* |  |
| **II. Outer setting domain** |  |  |
| Local Attitudes | **Negative perception mindfulness**  *"But it is a subject that is not yet embraced by everyone as a matter as something that should be part of a treatment strategy for a condition” - Healthcare insurer*  **Mindfulness not seen as part of healthcare**  *“But that doesn't make me think differently, because I think that with mindfulness you are concerned that you are medicalizing it.” - Healthcare insurer*  **Negative perception of the importance within medical field**  *“Yes, and then some gastroenterologists simply don't. They say yes, big nonsense or –” - IBD nurse*  *“In the more medical field there will be more stigma” - Professional association* | **Popularity of mindfulness**  *“Mindfulness is of course a lot more popular, so that can help you. Mindfulness is also much more popular in the general population than CBT treatment with the consequences model. And that can help you with implementation of course.” - Professional association*  **Positive perception of the importance within medical field**  *“And I'm sure there are a number of IBD doctors who also find this very important.” - IBD nurse* |
| Partnerships & connections |  | **Nationwide availability of teachers**  *“I say there are mindfulness trainers who really want that work. Then I would say, hey, make use of the knowledge that is already there” - Professional association* |
| Policies & laws | **Organizational policy of health insurers, Dutch Healthcare Authority, and Dutch Healthcare Institute does not align with innovative capacity**  *“The complexity lies in the fact that the way we currently organize, fund and regulate healthcare does not match the innovative capacity and need for innovation that we now have. That's my core problem. It assumes that we have one or two innovations per year nationally that we will carefully consider what to do with them.” - Healthcare insurer* |  |
| Financing | **No insurance coverage**  *“I do think that the costs are a barrier. Because it is not always insured.” – GP*  **No budget for innovative treatments**  *“Cause if we pay for this mindfulness training from healthcare funds, what are we going to stop with? It’s not that the budget gets bigger.” - Healthcare insurer* |  |
| External pressure - Societal pressure |  | **Attention to mindfulness via social media**  *“I think that it is now also gradually, for example within the Crohn's and Colitis Association, and so it* [mindfulness] *is coming to light in communication, on social media. There are also Facebook groups, closed groups within* [hospital name] *that we are of course not a member of, but where a lot of things are discussed.” – IBD nurse*  **Attention to lifestyle (outside healthcare)**  *“Sometimes there is something included in an additional coverage from an insurer because mindfulness is of course a theme that appeals, also to the insured” – Researcher in the field* |
| External pressure - Market pressure |  | **Attention to lifestyle in healthcare**  *“And there is currently much more attention to quality of life within IBD care. You see it on all sides in terms of nutrition and exercise and - So it wouldn't surprise me at all that this will also be included in this” - Gastroenterologist* |
| **III. Inner setting domain** |  |  |
| Structural characteristics |  |  |
| 1. Information technology infrastructure |  | **Digitization of healthcare provides time**  *“Now maybe they can fill that in themselves in HIX, I'll just name a few. So things will change, which may give you more time for such topics as in the case of mindfulness.” - IBD nurse* |
| 2. Work infrastructure | **Focus on production results in high workload and little time**  *“But yes, that is very difficult […]. But it is really production that we have to run here as well”* – IBD nurse  **Long waiting lists for mental healthcare in hospital**  *“You sometimes think, ‘hey, don’t you need some help?’ but then I’m already struggling with the fact that we do have a medical psychology department, but the waiting list is nine months” – Gastroenterologist*  **Pressure on healthcare providers**  *“But yes, in our current practice, and in many practices, the GP-mental health professionals are already overloaded.“ - GP* | **Perception of the role of healthcare provider**  *“Certainly. So that's the advantage, that it's a nursing theme. And that care belongs there, so to speak.” - IBD nurse*  **The presence of a local champion**  *“I think the reason we do it at the outpatient clinic is purely personal interest and that we have a personal interest in you wanting to stick your neck out” -Gastroenterologist* |
| Relative priority | **Focus/priority on medical aspect by healthcare professionals**  *“I think that there are also a lot of processing problems and existential problems, life stage problems that can occur with any chronic condition, which of course IBD also is. Yes, where we don't have much to offer, on average as an IBD practitioner [...] These are the areas where we as practitioners often neglect it.” – Professional association* |  |
| Mission alignment | **Hospital organizational policy focused on somatic care**  *“Unfortunately, we have phased out medical psychology with us” – Professional association* | **Hospital organizational policy focused on well-being**  *“Healthy well-being. And there is a lot of attention for this in the* [hospital name]*” - Mindfulness teacher* |
| Available resources - Space | **No suitable space**  *“There are a very limited number of group rooms in the house to sit with patients. Very real to be able to do those mindfulness exercises. You can't just take them to a meeting room.” - Medical Psychologist* |  |
| Available resources - Personnel | **Not enough personnel**  *“It’s not that the budget gets larger or that we suddenly have more healthcare professionals that can teach people mindfulness […] cause then you do not even have the professionals”* - H*ealthcare insurer* |  |
| Access to knowledge & information | **No knowledge on existing regional partners for mindfulness**  *“Well, if you know where that should go, you can just refer someone. I mean, you must agree with the patient where he or she can pick that up, so to speak. I really have no idea about that right now.” - IBD nurse* |  |
| **IV. Individuals domain** |  |  |
| Need | **Negative patient attitude**  “*Yes, that will be very variable. If you have the down-to-earth guy from North Holland, from the Lutjesbroek area, he might think what are you talking about?” - IBD nurse* | **Positive patient attitude**  *“What we mainly get back from people is that many people with IBD also practice mindfulness. It is often mentioned next to yoga, for example” - Patient representative*  **Ask about non-drug treatment**  *“There is increasing demand for what can I do myself outside of regular care, let's call it that. Outside of drug treatment.” - Patient representative*  ***Mindfulness meets patients’ needs***  *"Yes. No but I think there. I see merit in that. But I don't know if every manager sees that.” - IBD nurse* |
| Capability | **Difficult consultation – mental health**  “*Just like IBD patient groups that suffer very psychologically from their illness, most find it very nice and useful if attention is paid to this. We just don't like doing it ourselves.” – Professional association* | **(Perceived) Self-efficacy**  *“So in that sense I certainly feel qualified to have that conversation.” – GP*  *We zijn in de praktijk ook steeds meer bezig met positieve gezondheid. Ik denk dat we met z'n allen steeds meer en meer aandacht hebben voor de impact wat psyche op lichamelijke klachten kan hebben. Dus in die zin voel ik me er zeker toe bekwaam om dat gesprek aan te kunnen gaan – GP*  **Good relationship between doctor and patient**  *“So, what I do think is that these kinds of things, these kinds of interventions, also depend on the connection you have as a practitioner with your patient. I'm also a doctor by training, and I'm a medical advisor. And whether you're a doctor with a patient or an IBD nurse with a patient, I think the best thing, yes, is for your treatment relationship to sense where there's room for someone to do something. And in such a treatment relationship, you can sometimes offer something that sounds a bit strange.” -* Healthcare insurer |
| Opportunity | **Time consultation with doctor and IBD nurse**  *“But actually, they* [gastroenterologists] *have the least amount of time, so also less time to just have that good conversation” – Researcher in the field*  **Little access into patient group**  *“Well, I don't think so because I don't have that many IBD patients. They are often in the second line” - GP* | **Time for consultation with IBD nurse**  *“and perhaps it is also a little more important for the IBD nurse who simply has half an hour to go into all kinds of facets, that they are more likely to use this.” - Gastroenterologist* |
| Motivation |  | **Patient more motivated since training is adapted to target group**  *“Yes, and I think it might also help some participants: hey, this is something that is being investigated for our target group. And we feel extra invited to participate in that.” - Mindfulness teacher*  **Patient's willingness to pay**  *“There are of course a lot of people who try all kinds of things. Orthomolecular doctor and things. Hey, so people are quite willing to pay a lot to feel better” – Professional association* |
| **V. Implementation process domain** |  |  |
| Assessing needs | **Business case**  *“That still requires evidence, and I think it requires stories, especially from patients who participated and talk about what it has done for them. So, in follow-up research or mindfulness training, feedback. Yes, and scientific evidence. Yes, that would be fantastic, of course. Yes, if you can demonstrate a business case that, so to speak, fewer outpatient visits or fewer visits to a specialist take place there. Yes, that is—But that is unfortunately the harsh reality in a hospital.” – Mindfulness teacher*  **Broad support**  *“And I think it's actually almost something like, can you at some point convince the medical practitioner that mindfulness can also contribute to improving the health of these people?” – Professional association*  **Champions**  *“I think it's very important how you organize it. You'll need a champion” – Patient association*  **Funding**  *“The most important thing is of course that it will be reimbursed by the health insurer” - Gastroenterologist* | |
